# 2D/3D Non-Rigid Image Registration via Two Orthogonal X-ray Projection Images for Lung Tumor Tracking

**DOI:** 10.3390/bioengineering10020144

**Published:** 2023-01-21

**Authors:** Guoya Dong, Jingjing Dai, Na Li, Chulong Zhang, Wenfeng He, Lin Liu, Yinping Chan, Yunhui Li, Yaoqin Xie, Xiaokun Liang

**Affiliations:** 1 School of Health Sciences and Biomedical Engineering, Hebei University of Technology, Tianjin 300130, China; 2 Hebei Key Laboratory of Bioelectromagnetics and Neural Engineering, Tianjin 300130, China; 3 Tianjin Key Laboratory of Bioelectromagnetic Technology and Intelligent Health, Tianjin 300130, China; 4 Shenzhen Institute of Advanced Technology, Chinese Academy of Sciences, Shenzhen 518055, China; 5 Department of Biomedical Engineering, Guangdong Medical University, Dongguan 523808, China

**Keywords:** 2D/3D registration, orthogonal X-ray, deep learning

## Abstract

Two-dimensional (2D)/three-dimensional (3D) registration is critical in clinical applications. However, existing methods suffer from long alignment times and high doses. In this paper, a non-rigid 2D/3D registration method based on deep learning with orthogonal angle projections is proposed. The application can quickly achieve alignment using only two orthogonal angle projections. We tested the method with lungs (with and without tumors) and phantom data. The results show that the Dice and normalized cross-correlations are greater than 0.97 and 0.92, respectively, and the registration time is less than 1.2 seconds. In addition, the proposed model showed the ability to track lung tumors, highlighting the clinical potential of the proposed method.

## 1. Introduction

Medical imaging has helped a lot with diagnosing and treating diseases as modern medical technology has grown quickly. Image registration is crucial in medical image processing because it helps predict, diagnose, and treat diseases. For the images to be registered, three-dimensional (3D) medical images with rich anatomical and structural information are an inevitable choice for clinical problems. Unfortunately, 3D images have a higher radiation dose and a slower imaging speed, which inconveniences real-time clinical problems, such as image-guided radiotherapy and interventional surgery. On the other hand, two-dimensional (2D) images lack some spatial structure information, while the imaging speed is very fast. Therefore, in recent years, 2D/3D image registration with faster speed and simple imaging equipment has attracted much attention. The types of 2D images are usually X-ray [1,2,3,4], fluoroscopic [5], digital subtraction angiography (DSA) [6,7], or ultrasound [8], whereas 3D images are chosen from computed tomography (CT) [1,2,3,4] or magnetic resonance imaging(MRI) [8].

2D/3D registration methods can be divided into traditional and deep learning-based image registration. In traditional image registration, 2D/3D alignment usually translates into the problem of solving for the maximum similarity between digitally reconstructed radiographs (DRR) and X-ray images. Similarity metrics are usually based on intensity-based mutual information [9,10,11], normalized cross-correlation (NCC) [12] and Pearson correlation coefficients [13], or gradient-based similarity metrics [14]. To minimize the dimensionality of the transformation parameters, regression models that rely on a priori information are usually built using B-spline [15] or principal component analysis (PCA) [16,17,18,19]. However, organ motion and deformation can cause errors in regression models, which rely too much on prior information. By incorporating finite element information into the regression model, Zhang et al. [18,19] obtained more realistic and effective deformation parameters. However, adding finite element information makes the model-driven method of finding the optimal solution iteratively more inefficient. Therefore, this process is a constraint for developing real-time 2D/3D registration and tumor tracking algorithms.

With the development of artificial intelligence and deep learning, learning-based methods replace the tedious iterative optimization process with predicted values in the testing process, greatly improving computing efficiency. Zhang [20] proposed an unsupervised 2D-3D deformable registration network that addresses 2D/3D registration based on finite angles. Li et al. [4] proposed an unsupervised multiscale encode decode framework to achieve non-rigid 2D/3D registration based on a single 2D lateral brain image and 3D CBCT image. Ketcha et al. [21] used multi-stage rigid registration based on convolutional neural networks (CNN) to obtain a deformable spine model. Finally, Zhang et al. [22] achieved a deformable registration of the skull surface. Unfortunately, the above learning-based approach evaluates the similarity between DRR and X-ray, a 2D/3D registration reduced dimension to 2D/2D registration. Therefore, it is inevitable that spatial information will be lost to some extent. In addition, even with Graphic Processing Unit (GPU) support, forward projection, backward projection, and DRR generation involved in the above methods are computationally expensive. Then, the researchers completed end-to-end 2D/3D registration by integrating the forward/inverse projection spatial transformation layer into a neural network [3,23]. Frysch et al. [2] used Grangeat’s relation instead of expensive forward/inverse projection to complete the 2D/3D registration method based on a single projection of arbitrary angle, which greatly accelerated the computational speed. However, this is a rigid transformation which is difficult to apply to elastic organs. Likewise, deep learning researchers have attempted to use statistical deformation models to build deep learning-based regression models. Using a priori information to build patient-specific deformation spaces, convolutional neural networks are used to accomplish regression on PCA coefficients [1,24,25] or B-spline parameter coefficients [26,27] to achieve patient-specific registration networks. Tian et al. [28] obtained the predicted deformation field based on the regression coefficients. However, this deformation space, which is completely based on a priori information, may lead to mistakes in the clinical application stage. In addition, some researchers [29,30] also accomplished 2D/3D image registration by extracting feature points. With the maturity of point cloud technology, many researchers have also built point-to-plane alignment models by extracting global point clouds to complete 2D/3D alignment models, but the anomaly removal for 2D/3D alignment models presents a challenge [31,32,33]. Graphical neural networks are also used for 2D/3D registration in low-contrast conditions [34]. Shao et al. [35] tracked liver tumors by adding finite element modeling. Still, the introduction of finite elements also brought some trouble to the registration time.

Therefore, we developed a deep learning-based method for non-rigid 2D/3D image registration of the same subject. Compared with traditional algorithms based on iterative optimization, this approach significantly improves the registration speed. Compared with the downscaled optimization of DRR and X-ray similarity, we optimized the similarity of 3D/3D images, which can effectively moderate the loss of spatial information. Additionally, only two projections based on orthogonal angles were chosen for 2D images to reduce the irradiation dose further. The proposed method is used to study the process of changes in the elastic organ as respiratory motion proceeds. More significantly, we also investigated the change in tumor position with respiratory motion, which can be used to achieve tracking of tumors based on orthogonal angular projections during radiotherapy.

The contributions of our work are summarized as follows:

1. We propose a 2D/3D elastic alignment framework based on deep learning, which can be applied to achieve organ shape tracking at lower doses using only two orthogonal angles of X-rays.

2. Our framework is expected to be used for tumor tracking with tumor localization accuracy up to 0.97 and registration time within 1.2 s, which may be a potential solution for image-guided surgery and radiotherapy.

The organizational structure of this article is as follows. Section 2 describes the experimental method. Section 3 describes the experiment setups. Section 4 shows the Result. Section 5 is the discussion and Section 6 concludes the paper and the references.

## 2. Methods

### 2.1. Overview of the Proposed Method

**Figure 1:**
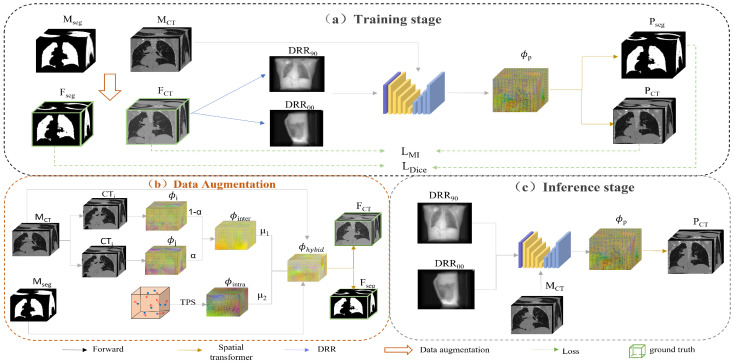
Overview of the proposed method. (**a**) Flowchart of the training phase of the method. First, a large number of CT $F_{CT}$ and segmentation $F_{seg}$ representing each phase are obtained by performing hybrid data augmentation of the moving image $M_{CT}$ and the corresponding segmentation image $M_{seg}$. Then, the $F_{CT}$ images are projected to obtain the 2D $DRR_{90}$ and $DRR_{00}$. After that, they are fed into the registration network with the moving image $M_{CT}$ to obtain the predicted deformation field $\phi_{p}$. Finally, the moving image $M_{CT}$ and the moving segmentation map $M_{seg}$ are transformed to obtain the corresponding predicted images, $P_{CT}$ and $P_{seg}$. (**b**) The process of hybrid data augmentation. The deformation field $\phi_{inter}$ is first obtained by inter-phase registration using traditional image registration. The small deformation $\phi_{intra}$ is simulated by TPS interpolation. The hybrid deformation field $\phi_{hybrid}$ is obtained by summing with random weights for data augmentation. (**c**) Inference stage. The 2D projection and moving images are directly input to the trained network to get the prediction $\phi_{p}$, and then the registration can be completed by transformation.

The framework of this method is shown in Figure 1. We design a non-rigid 2D/3D registration framework based on deep learning of orthogonal angle projection. Since it is a deep learning-based model, a large amount of data is needed to participate in training. The real paired 2D/3D medical images at the same time are very scarce, so the first task that needs to be done is data augmentation. We chose 4D CT of the lungs as the experimental subject. The expiratory end was used as a moving image $M_{CT}$ and hybrid data augmentation [36,37] was used to obtain a large number of CT $F_{CT}$ representing each respiratory phase of the lung (this procedure will be described in Section 3.1). Then, the ray casting method obtains a pair of 2D DRRs of $F_{CT}$ with orthogonal angles. After that, the orthogonal DRR and the moving image $M_{CT}$ are input into the 2D/3D registration network. The network outputs a 3D deformation field $\phi_{p}$. Then, the moving image $M_{CT}$ is transformed by the spatial transformation layer [38] to obtain the corresponding predicted CT image. The maximum similarity between the predicted CT image and the ground truth $F_{CT}$ is calculated. Through continuous iterative optimization, we can complete the model training. In the inference phase, only the X-ray projections or DRRs and the moving image must be input to the trained network to get corresponding 3D images.

### 2.2. 2D/3D Registration Network

**Figure 2:**
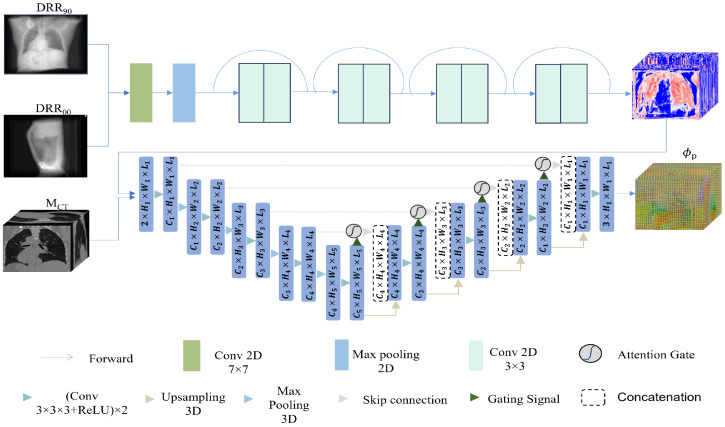
2D/3D registration network. First, 2D DRRs at orthogonal angles are processed by residual blocks to obtain 3D feature maps. Then, the feature maps and moving images are fed into a 3D Attu-based encode–decode network. The final output of this network is the predicted 3D deformation field.

Figure 2 shows the registration network. For 2D/3D image registration, the first thing to consider is the consistency of spatial dimension. As a result, we use the extracted feature up-dimensional approach to transform the 2D/3D registration problem into the 3D/3D registration problem. We used the residual network to get the 2D features. The most important step is identity mapping, stopping the gradient from going away, and helping train the network. Thus, when two DRRs with orthogonal angles are input to the network, they are first concatenated in the channel layer as the input of the residual network and then passed through the convolution layer, the max pooling layer, and two output channels with 64 and 128 residual blocks in turn. The channel layer is the third dimension to form a 3D feature map, which is input to the feature extraction network together with the moving image.

We selected the 3D Attention-U-net [37,39] (3D Attu) as the feature extraction network in this study. It can be called the 3D/3D matching network. The network 3D Attu adds an attention gate mechanism to the original U-net, which can automatically distinguish the target shape and scale, and learn more useful information. It also employs encoding and decoding mechanisms and skips connection mechanisms. It effectively blends high- and low-level semantic information while widening the perceptual domain. It has been used in many medical image processing tasks with excellent results. As a result, in this model, we feed the moving image $M_{CT}$ and the 3D feature map into the 3D Attu. The output is the predicted deformation field.

### 2.3. Loss Function

The mutual information (MI) between the ground truth $F_{CT}$ and the predicted 3D CT $P_{CT}$ obtained by the registration network constitute the loss function $L_{MI}\left(F_{CT},P_{CT}\right)$. The other part of the loss function is $L_{Dice}\left(F_{seg},P_{seg}\right)$, obtained by computing the Dice between the corresponding segmented images, which allows the model to focus more on the lung region. Lastly is the regularized smoothing constraint $L_{Reg}\left(\phi_{p}\right)$ for the deformation field.

(1) $$L_{Dice}\left(F_{seg,}P_{seg}\right)=\sum_{i=0}^{n}\frac{1}{n}\frac{2\left|F_{seg}^{i}\cap P_{seg}^{i}\right|}{\left|F_{seg}^{i}+P_{seg}^{i}\right|}$$

(2) $$\begin{array}{c} L=\lambda_{1}L_{Dice}\left(F_{seg,}P_{seg}\right)+\lambda_{2}L_{MI}\left(F_{CT},P_{CT}\right)+\lambda_{3}L_{Reg}\left(\phi_{p}\right) \end{array}$$

where *n* denotes the number of categories in the image, *i* denotes the *i*-th category of the image. φ denotes all elements in the entire deformation field. $\lambda_{1}$, $\lambda_{2}$, $\lambda_{3}$ denote the weights of $L_{Dice}$, $L_{MI}$, $L_{Reg}$ respectively, which were chosen as 0.5, 0.5, and 0.1 in this experiment.

## 3. Experiment Setups

### 3.1. Data and Augmentation

We conducted experiments on three different types of lung data, TCIA [40,41,42,43] patient with a tumor, Dirlab [44] lung CT without tumor, and CIRS phantom. ITK-SNAP is used for automatic segmentation to obtain labels. In the TCIA patient data, we selected one of the patients for the experiment. In Dirlab, we selected the first five sets of data for the experiment. In the CIRS phantom, we simulated the lung tumor with a water sphere. In the experiment, we resampled the 3D CT image to 128 * 128 * 128 with a voxel spacing of 1 mm * 1 mm * 1 mm. Since our experiment is a 2D/3D registration, paired 2D projections and 3D medical images of the same moment are rare. It is unethical to expose the human body to additional radiation doses, so the first task is data augmentation. However, for 2D/3D registration of the treatment phase (e.g., radiotherapy, surgical navigation), it is obvious that the focus is more on the specific person. Therefore, we chose a hybrid data augmentation approach to train a deep learning-based 2D/3D registration model for a specific human body.

In the hybrid data augmentation shown in Figure 1b, we first selected the end-expiratory phase of 4D CT as the moving image $M_{CT}$ and the remaining phases as the fixed image $CT_{1,\ldots ,i,j,9}$. Then, we used the conventional intensity-based image registration method to obtain nine deformation fields in the order of $\phi_{1,\ldots ,i,j,9}$. The deformation fields used for data augmentation were arbitrarily selected from two of the nine deformation fields and superimposed with random weights to obtain many inter-phase deformations. The lung may also change during respiratory motion. Therefore, we use thin plate spline (TPS) interpolation to simulate small changes in specific phases. The number of control points N was randomly chosen between 20 and 60. The movement distance of control points was chosen between 0 mm and 20 mm to obtain many phase-specific random deformations. To obtain more morphologically diverse images, we combined inter-phase and intra-phase specific deformation with random weights to obtain many hybrid deformation fields. Spatial warping of the moving and segmented images was performed to obtain CT and segmented images representing each respiratory phase of the lung.

### 3.2. DRR Image Generation

**Figure 3:**
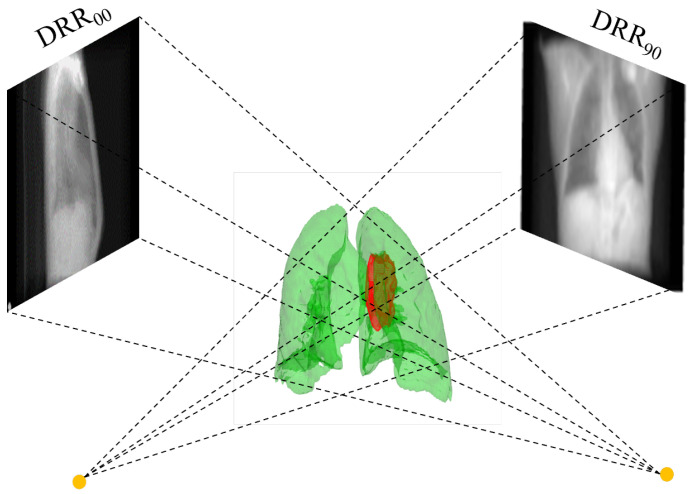
Schematic diagram of DRR image generation.

The orthogonal angle X-ray projection system in this experiment is shown in Figure 3. Two-point light sources at orthogonal angles emit rays through the object and project them on two detectors perpendicular to the central axis. We assume that the initial intensity of $I_{0}$ at the light source, μ is the internal attenuation coefficient of the object to the rays, *I* is the thickness of the ray through the object, and $I_{n}$ is the intensity of the ray after passing through the object. The formula $I_{n}=I_{0}e^{-\int \mu (l)dl}$ arises. After the projection of one ray is finished, the attenuation coefficient obtained by accumulating the whole path and then converting it to CT value is the X-ray image. In this experiment, like most researchers, DRR images with the same imaging principle are used instead of X-ray. Virtual X-rays were used to pass through the CT images, and after attenuation, they were projected onto the imaging plane to reconstruct the DRR images. The 3D CT images representing each respiratory phase after data augmentation are projected using this method to obtain the DRR images at the corresponding moment. This technique has been widely used for 2D/3D registration methods.

### 3.3. Experiment Detail

We used hybrid data augmentation to obtain 6000 samples from the three types of experimental data. Of these, 5400 were used as the training set, 300 as the validation set, and 300 as the test set. Our experiment was implemented using the deep learning framework Pytorch 1.10 on a NVIDIA A6000 GPU with 48 G of memory, and an AMD Ryzen 7 3700X 8-core processor with 128 GB of internal memory. The learning rate is set to $10^{-4}$. For all datasets, the batch size was set to 8 and the optimization algorithm is Adam.

### 3.4. Experiment Evaluation

In order to verify that our model can achieve 2D/3D registration by two orthogonal angular projections, we selected the end of expiration as the moving image and aligned it toward the remaining phases. We evaluated the three-lung data using NCC, MI, 95% Hausdorff surface distance, and Dice. In addition, to explore the tracking of lung tumors that can be achieved by our model, we compared between predicted and ground truth values for the dataset with tumors and quantitatively evaluated using Dice and the tumor center of mass.

## 4. Result

### 4.1. Registration from the Expiratory End to Each Phase

**Figure 4:**
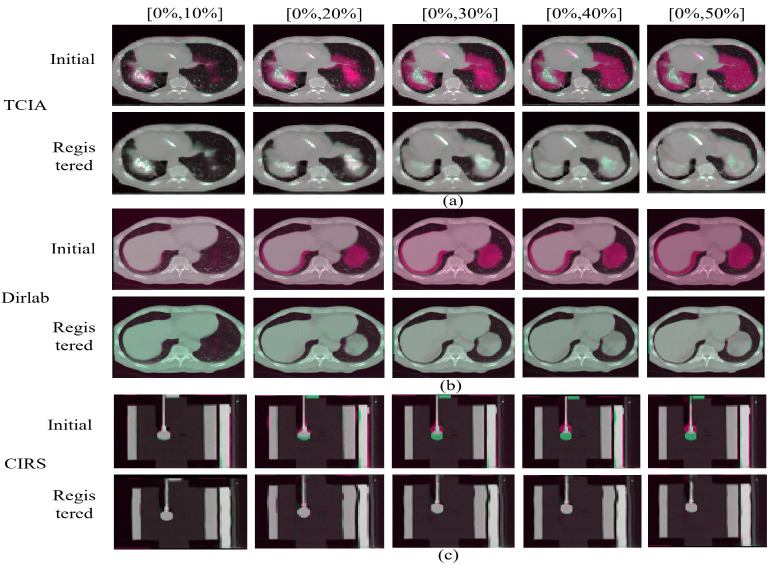
Registration from the exhalation end to the other stages. (**a**) shows the results of our registration on TCIA, (**b**) a randomly selected set of experiments from Dirlab, and (**c**) the registration results of the phantom data. The odd-numbered rows are the unregistered contrast images, and the even-numbered rows are the registered contrast images.

Here, we demonstrate the registration results of each phase from the end of expiration to the end of inspiration for the TCIA, Dirlab, and phantom. For the qualitative assessment, Figure 4a shows the results of our selected experiments on patients with tumors on TCIA, Figure 4b shows a randomly selected set of experiments from Dirlab, and Figure 4c shows the effect of registration of the phantom data. The odd rows are the unaligned ones, and the even rows are the aligned results. Based on the results, both TCIA patients with tumors and without tumors in Dirlab, as well as the phantom model with water balloons that simulate tumors, can achieve registration from the end of expiration to the rest of the stages.

For the quantitative analysis, we used Dice of the segmentation map, 95% Hausdorff surface distance, NCC, and MI of grayscale images to evaluate our scheme separately. The results are shown in Table 1. It can be seen that good registration results are obtained for all three types of data, not only on the grayscale images, but also on the lung of interest. The Dice values of all three data types are above 0.97, the Hausdorff surface distances are below 2 mm, NCC are above 0.92 and MI are above 0.90. Compared with the real human lung, the NCC and MI of the phantom data are relatively small because the lung of the phantom itself does not change. Only the internal water sphere changes, which is more rigidly transformed relative to the real patient, so the NCC and MI are relatively small at higher Dice. However, the total accuracies are still above 0.92 and 0.90. Therefore, quantitative and qualitative results show that the proposed method can achieve non-rigid 2D/3D registration for a specific subject by two orthogonal angular projections.

### 4.2. Tumor Location

Both TCIA patient and the phantom contained tumors. The accuracy of tumor localization was evaluated qualitatively and quantitatively.

**Figure 5:**
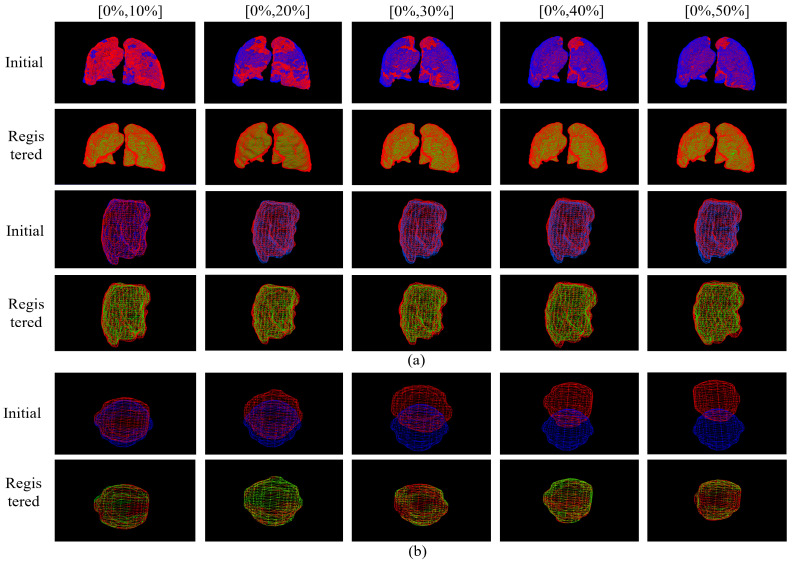
Tumor registration results from the exhalation end to other stages. Where (**a**) is the 3D presentation of the results before and after a real patient’s lung and tumor registration, and (**b**) is of the phantom data of the 3D results of the tumor display. The odd rows are the unregistered images, and the even rows are the post-registered images. The red image indicates the ground truth. Blue is the moving image, and green is the predicted result obtained by the model.

Figure 5 shows the qualitative evaluation of the 3D tumor with two types of data, where (a) is a 3D visualization image of the patient’s overall lung and tumor and (b) is of the phantom data. Table 2 presents the quantitative results, where we evaluated the tumor center mass and Dice. The tumor center of mass deviation is within 0.15 mm for the real patient. The phantom tumor center of mass is less than 0.05 mm. The Dice of both are above 0.88. It can be seen that the proposed method can achieve registration both for the whole lung and for the tumor. In addition, the fact that local tumors are well aligned suggests that our model could be useful for clinical applications such as tracking tumors.

## 5. Discussion

### 5.1. Traditional Registration in Data Augmentation

**Figure 6:**
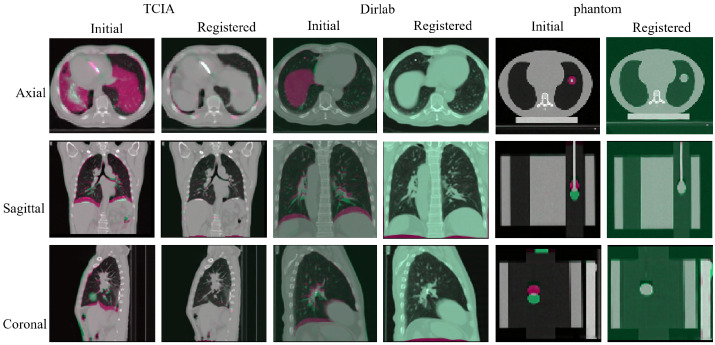
The traditional registration method results from the end of exhalation to the end of inhalation. The first two columns are the registration results on the patient, the middle two are the registration results on the normal human lung, and the last two are the registration results on the phantom. The odd columns are the unregistered images, and the even columns are the results of the registered images.

We used traditional intensity-based image registration for data augmentation to complete the registration between phases. Here we present the two most distorted parts of the three data, the end of expiration and the end of inspiration, for evaluation. The experimental results are shown in Figure 6.

Figure 6 shows the results from three directions before and after the registration. The odd columns are the unregistered images. The even columns are the results after the traditional registration method. Conventional image registration can be accomplished for real patients and models from exhalation to the end of inspiration, ensuring that our augmentation data encompasses all respiratory phases of the lung. In addition, the registration covers the larger deformations at both ends.

### 5.2. Landmark Error

For the Dirlab data, the landmark points and the deformation field for performing data augmentation are known. Thus, the landmark points of the image generated after data augmentation are also known and used as our ground truth. The mean target registration error (mTRE) is evaluated with our model-predicted images.

**Figure 7:**
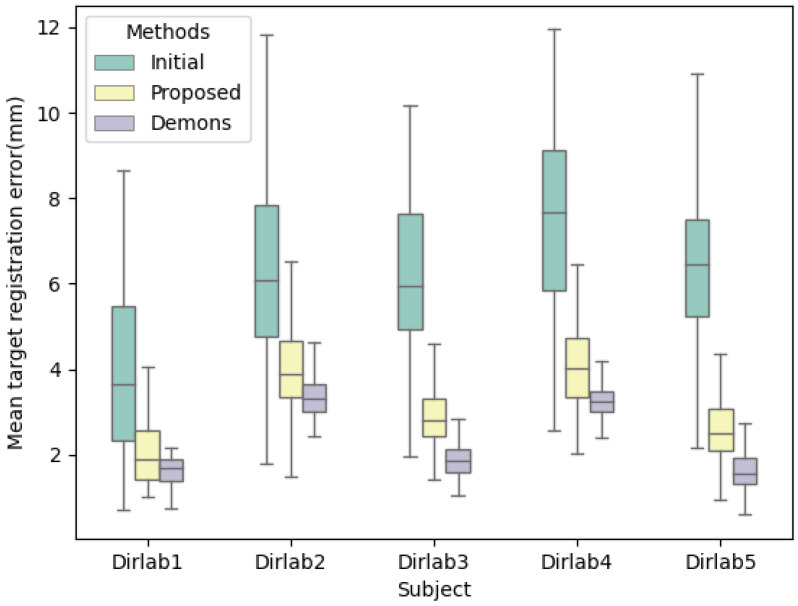
Box plot of landmark points in Dirlab; green indicates the data before registration, yellow is the data after registration of the proposed method, and purple represents the data after 3D/3D registration using Demons.

The data obtained from the evaluation are shown in Table 3. The corresponding box plot is shown in Figure 7, in which green indicates the data before registration, yellow is the data after registration of the proposed method, and purple represents the data after 3D/3D registration using Demons [45]. The results show that the proposed model can achieve effective 2D/3D registration, but the accuracy is lower than the existing advanced 3D/3D registration models because the experimental data are only two 2D X-rays with orthogonal angles. Although the proposed method transforms 2D/3D registration into a 3D/3D registration problem, some image details are indeed lost compared with the 3D images, resulting in the loss of information on tiny details, such as capillaries, leading to a lower accuracy of landmark error based on detailed information. However, in the 2D/3D registration mission, more attention is paid to the global overall changes in the lung and the tumor location. The proposed method greatly reduces the irradiation dose and improves the registration speed.

In addition, our method can complete 2D/3D registration in 1.2 s. In contrast, other data-driven 2D/3D registration models, such as [20], may take a few seconds. On the other hand, traditional image registration methods may take tens of minutes or even hours. Our method also only needs two different angles of X-rays, which greatly reduces the amount of radiation and makes the hardware in the clinic easier to use. Of course, our method also has some limitations. First of all, since real medical images do not exist at the same moment of paired orthogonal angles of X-ray and corresponding 3D CT, we use 2D DRR. Although DRR and real X-ray use the same imaging way, it is undeniable that there are some grayscale and noise differences between the two. However, it can be corrected by using existing methods, such as histogram matching [24], network of GAN [25], etc., which is not the main focus of our study. We will also make some improvements to the program to speed up the processing speed for radiotherapy or interventional procedures that require more real-time, etc. In addition, since there are few non-rigid 2D/3D registration articles, the code is not open source. We have yet to choose a suitable comparison experiment, and we will continue to look for it in the future.

## 6. Conclusions

This study proposes a deep learning-based 2D/3D registration method using two orthogonal angular X-ray projection images. The proposed algorithm has been verified on lung data with and without tumor and phantom data, and obtained high registration accuracy, where Dice and NCC are greater than 0.97 and 0.92. In addition, we evaluated the accuracy on the data containing tumor, and the tumor center-of-mass error was within 0.15 mm, which indicates the promising use of our model for tumor tracking. The registration time is within 1.2 s, and this is promising for clinical applications, such as radiotherapy or surgical navigation, to track the shape of organs in real time. Moreover, we only need to use two orthogonal angles of X-rays to achieve 2D/3D deformable image registration, which can greatly reduce the extra dose during treatment and simplify the hardware system required. 

## Figures and Tables

**Table 1:** The accuracy of subjects’ registration from the expiratory end to each phase.

		Dice	Hauf (95%)	NCC	MI
TCIA	[0%,10%]	0.9814	1.1210	0.9846	0.9796
	[0%,20%]	0.9791	1.3811	0.9846	0.9760
	[0%,30%]	0.9795	1.3811	0.9847	0.9540
	[0%,40%]	0.9785	1.3811	0.9846	0.9578
	[0%,50%]	0.9806	1.4196	0.9848	0.9650
Dirlab	[0%,10%]	0.9857	1.8839	0.9762	0.9590
	[0%,20%]	0.9857	1.8620	0.9723	0.9535
	[0%,30%]	0.9853	1.8280	0.9691	0.9511
	[0%,40%]	0.9853	1.8290	0.9680	0.9424
	[0%,50%]	0.9854	1.8290	0.9753	0.9608
CIRS	[0%,10%]	0.9862	0.9043	0.9338	0.9135
	[0%,20%]	0.9907	1.6713	0.9291	0.9023
	[0%,30%]	0.9888	2.0000	0.9360	0.9064
	[0%,40%]	0.9885	1.9087	0.9348	0.9065
	[0%,50%]	0.9894	1.9087	0.9349	0.9178

**Table 2:** The accuracy of tumor location from the expiratory end to each phase.

		Center Mass (mm)				Dice
		X(LR)	Y(AP)	Z(LR)	Center	Tomor
TCIA	[0%,10%]	0.0003	0.0473	0.0844	0.0968	0.9440
	[0%,20%]	0.0117	0.0133	0.0260	0.0315	0.9434
	[0%,30%]	0.0031	0.0023	0.0473	0.0022	0.9023
	[0%,40%]	0.0032	0.0078	0.0339	0.0350	0.9080
	[0%,50%]	0.0227	0.0510	0.1251	0.1370	0.8984
CIRS	[0%,10%]	0.0224	0.0011	0.0270	0.0351	0.9717
	[0%,20%]	0.0061	0.0025	0.0081	0.0104	0.9764
	[0%,30%]	0.0086	0.0018	0.0158	0.0181	0.9609
	[0%,40%]	0.0174	0.0177	0.0084	0.0262	0.9702
	[0%,50%]	0.0022	0.0043	0.0477	0.0479	0.8826

**Table 3:** Mean target registration error of landmarks in Dirlabs.

(mm)	Initial	Proposed (2D/3D)	Demons (3D/3D)
Dirlab1	3.9776 (1.8616)	2.0065 (0.6748)	1.6297 (0.3196)
Dirlab2	6.3989 (2.1719)	4.0079 (1.0077)	3.3807 (0.5089)
Dirlab3	6.2138 (1.7843)	2.9219 (0.7237)	2.1556 (0.2991)
Dirlab4	7.6437 (2.3978)	4.0682 (0.9898)	3.2525 (0.3918)
Dirlab5	6.6075 (2.1448)	2.6253 (0.7719)	1.6408 (0.4824)

## Data Availability

The dataset images used for this study is publicly available on TCIA at https://wiki.cancerimagingarchive.net/pages/viewpage.action?pageId=21267414 (accessed on 27 October 2022) and Dirlab at https://med.emory.edu/departments/radiation-oncology/research-laboratories/deformable-image-registration/downloads-and-reference-data/4dct.html (accessed on 27 October 2022).

## Abbreviations

The following abbreviations are used in this manuscript:

2D	Two-dimensional
3D	Three-dimensional
DSA	Digital Subtraction Angiography
CT	Computed Tomography
MRI	Magnetic Resonance Imaging
DRR	Digitally Reconstructed Radiographs
NCC	Normalized cross-correlation
PCA	Principal Component Analysis
CNN	Convolutional Neural Network
GPU	Graphic Processing Unit(GPU)
3D Attu	3D Attention-U-net
MI	Mutual Information
TPS	Thin plate spline
mTRE	Mean Target Registration Error
